# Evolving Dynamics of Neck Muscle Activation Patterns in Dental Students: A Longitudinal Study

**DOI:** 10.3390/s24175689

**Published:** 2024-09-01

**Authors:** Manuel Barbosa de Almeida, Marion Moreira, Paulo Miranda-Oliveira, José Moreira, Carlos Família, João R. Vaz, Paula Moleirinho-Alves, Raúl Oliveira

**Affiliations:** 1 Neuromuscular Research Lab, Interdisciplinary Centre for the Study of Human Performance (CIPER), Faculty of Human Kinetics, University of Lisbon, Oeiras, 1499-002 Lisbon, Portugal; mbalmeida@egasmoniz.edu.pt; 2 Egas Moniz Center for Interdisciplinary Research (CiiEM), Egas Moniz School of Health & Science, Caparica, 2829-511 Almada, Portugal; 3 Department of Physiotherapy, Egas Moniz School of Health & Science, Campus Universitario, Quinta da Granja, Caparica, 2829-511 Almada, Portugal; 4 ESTG–School of Technology and Management, Polytechnic Institute of Leiria, 2411-901 Leiria, Portugal; 5 Performance, Research and Planning Department, Portuguese Athletics Federation, 2799-538 Linda-A-Velha, Portugal; 6 Nursing School São João de Deus, University of Évora, 7004-516 Évora, Portugal; 7 Comprehensive Health Research Centre, National School of Public Health, 1600-560 Lisbon, Portugal

**Keywords:** cervical pain, dentistry students, electromyography, cranio-cervical flexion test, activation pattern, motor control

## Abstract

Cervical pain has been linked to increased motor unit activity, potentially associated with the initiation and progression of chronic neck pain. Therefore, this study aimed to compare the time-course changes in cervical superficial muscle activation patterns among dental students with and without neck pain throughout their initial semester of clinical training. We used an online Nordic Musculoskeletal Questionnaire for group allocation between neck pain (NP) (*n* = 21) and control group (CG) (*n* = 23). Surface electromyography (sEMG) of the sternocleidomastoid and upper bilateral trapezius was recorded before starting their clinical practice and after their first semester while performing a cranio-cervical flexion test (CCFT) in five increasing levels between 22 mmHg and 30 mmHg. After the first semester, both the CG (*p* < 0.001) and NP (*p* = 0.038) groups showed decreased sternocleidomastoid activation. The NP group exhibited a concomitant increase in upper trapezius coactivation (*p* < 0.001), whereas the muscle activation pattern in asymptomatic students remained unchanged (*p* = 0.980). During the first semester of clinical training, dental students exhibited decreased superficial flexor activity, but those with neck pain had increased co-contraction of the upper trapezius, likely to stabilize the painful segment. This altered activation pattern could be associated with further dysfunction and symptoms, potentially contributing to chronicity.

## 1. Introduction

Neck pain stands as a globally recognized contributor to disability, affecting approximately 4.82% of the population and ranking as the fourth leading cause of disability worldwide [1,2]. In professions characterized by heightened cervical strain, such as dentistry, the prevalence of neck pain is up to 51% of dental students [3], even in the early training phases. This rate significantly surpasses the general population’s reported rate by over tenfold [1,2] and triples the reported rates within the broader working population [4]. This could be associated with significant cervical impairment and functional deficiencies, which reflect altered mechanisms of muscle control and changed muscle properties [5]. While the basic physiological mechanisms of pain have been extensively studied [6,7,8] and the functional impairments [6] associated with neck pain are well documented, the relationships between neck pain and motor control are still poorly understood [6,9]. This is primarily due to the challenge of translating basic physiological findings into the complex clinical context of pain conditions [6]. Pain, inherently multifactorial, manifests through unique experiences that induce specific motor responses and adaptations [7,8]. Cervical pain has been linked to increased motor unit activity [10,11,12,13,14,15], which could play a crucial role in the potential initiation, development, and progression of chronic neck pain [12,16]. However, despite the established connections among pain, motor control, and muscle properties, their specific contributions in perpetuating neck pain and its recurrence still need to be explored [6]. The activation of nociceptors profoundly affects both spinal and supraspinal pathways that regulate muscle control [5]. It also indirectly affects muscle spindle sensitivity through sympathetic nervous system activation, and the pain modifies the descending neural drive from supraspinal centers [5]. Electrophysiological methods, such as surface electromyography (sEMG), enable the examination of muscle activity and coordination and a greater understanding of the complex interactions at the end of these nociceptive pathways. Given the high prevalence of this condition among dental students, it is imperative to evaluate the specific neuromuscular adaptations associated with their clinical practice and setting [5,6,9].

Therefore, this study aimed to compare the time-course changes in cervical superficial muscle activation patterns among students with and without neck pain throughout their initial semester of clinical training.

## 2. Materials and Methods

### 2.1. Study Design

This longitudinal study obtained approval from the Egas Moniz School of Health & Science Ethics Committee (Approval No. CEEM-1122, September 2022). To uphold participant confidentiality, all data were anonymized, following the European Union General Data Protection Regulation (GDPR). The scheduled deletion of these data is set for December 2028. The study’s methodologies were crafted to comply with the ethical standards outlined in the 1975 Declaration of Helsinki (as amended in 2008). Furthermore, the design and reporting of our findings strictly adhered to the guidelines outlined in the Strengthening the Reporting of Observational Studies in Epidemiology (STROBE) statement [17].

### 2.2. Participants

Fourth-year dental students in the master’s degree program at Egas Moniz School of Health & Science, Portugal, voluntarily participated in this study. Participants were assigned to one of two groups based on their Nordic Musculoskeletal Questionnaire (NMQ) responses: the asymptomatic students for the control group (CG) and fourth-year students with neck pain (NP). The allocation of groups was concealed from all investigators and assessors by keeping the file hidden until the completion of the initial assessment. Inclusion criteria were (i) students actively involved in clinical practice and (ii) students from the 4th year. Exclusion criteria were (i) students who had clinical practice before September 2023, (ii) over 30 years of age, (iii) students with ongoing musculoskeletal disorders, (iv) inability or unwillingness to provide informed consent, (v) communication difficulties with investigators, (vi) major neurological disorders, (vii) dysfunctions of the central or peripheral nervous system, (viii) diagnosed schizophrenia, bipolar disorder, or other psychotic disorders, (ix) alcohol or other substance abuse, and (x) major depression.

A minimum sample size of 14 participants per group was determined using G*Power software (v.3.1.9.7, Düsseldorf University, Düsseldorf, Germany), accounting for an α of 0.05 (5% chance of Type I error), a minimum power (1-β) of 0.95, and a large effect size of 0.361 [18].

### 2.3. Procedure

The student’s group allocation was performed using an online form with informed consent and the Nordic Musculoskeletal Questionnaire (NMQ) [19]. The questionnaire was administered on two occasions: initially at the beginning of the academic year in September and after 15 weeks at the end of the semester in December. Before participating, each participant was required to read and provide their consent digitally before proceeding to complete the questionnaire and the subsequent surface electromyography assessment.

#### 2.3.1. Electromyography Data Acquisition

Surface electromyography data were recorded for the superficial neck flexors and extensors according to established protocols [20,21], following the recommendations of the Surface Electromyography for the Non-Invasive Assessment of Muscles (SENIAMs). The skin was shaved and cleaned with 70% alcohol before electrode placement to ensure sEMG signal quality. Disposable Ag/AgCl Ambu^®^ BlueSensor N electrodes (Ambu A/S, Ballerup, Denmark) were utilized, each with a 10 mm diameter and positioned 20 mm apart from center-to-center. Electrodes were applied bilaterally over the sternal head of sternocleidomastoid (SCM), on the distal third of the muscle belly to avoid the innervation zone [21], and over the upper trapezius (UT) muscle on the muscle belly at a midpoint between C7 spinous process and the acromial process [20]. The reference electrode was placed over the center of the clavicle bone. Surface electromyography data were amplified and digitized using the biosignalsplux 8-channel hub (PLUX Wireless Biosignals S.A., Lisbon, Portugal), featuring an analog-to-digital converter with a resolution of 16 bits per channel, input impedance exceeding 100 GOhm, an amplifier gain of 1000, a common-mode rejection ratio of 100 dB, and with a sampling frequency of 1000 Hz.

#### 2.3.2. Maximum Voluntary Isometric Contraction (MVIC)

For the MVIC record, participants were supine with arms alongside the body, and they were instructed to perform a combined head and neck flexion able to gently lift the head off the plinth, maintaining this position against the examiner’s manual resistance applied at the forehead’s midline for two seconds [22]. For neck extension, participants were instructed to perform the neck extension for two seconds against the plinth in the same position. Surface electromyography data of MVIC was collected in a single file integrating flexion and extension measurements for sEMG normalization purposes.

#### 2.3.3. Cranio-Cervical Flexion Test (CCFT)

Clinical evaluations were conducted following established procedures [13,22,23] using the cranio-cervical flexion test (CCFT) with a blinded physiotherapist overseeing the process. During the procedure, participants were positioned supine on a physiotherapy plinth. At the same time, an inflatable air-filled pressure sensor (Stabilizer^®^, Chattanooga, South Pacific, TN, USA) was placed behind their neck and inflated to 20 mmHg. Participants were instructed to engage in a gradual head-nodding motion, specifically targeting the upper cervical spine, and increase the pressure in 2 mmHg increments through five stages, from 22 mmHg to 30 mmHg [13,22,23]. Before the formal testing, subjects underwent a familiarization phase preceding the actual CCFT to correct and discourage improper techniques, such as head retraction, lifting, or post-movement tension. Following this phase, participants were provided a one-minute rest period before commencing the formal CCFT assessment. The formal test consisted of maintaining each pressure level for 10 s, with 30 s of rest between stages [13,22,23]. Surface electromyography was concurrently recorded from the superficial neck flexors and extensors, as detailed subsequently.

#### 2.3.4. Electromyography Data Processing

The sEMG signals were wirelessly transmitted to a laptop (AMD Ryzen 5, 8 GB RAM, 500 GB SATA HDD, Windows 11 64-bit operating system) with Opensignals^®^ software (v.2.2.5, Plux Wireless Biosignals S.A., Lisboa, Portugal) to monitor and store the retrieved data. A 10-s recording from the four channels was saved in a single file for each participant and level of the CCFT. Electromyographic signals were preprocessed with Matlab (v.R2023b, The Mathworks Inc., Natick, MA, USA) with a customized script based on a standard procedure [24] to ensure signal quality and integrity during recordings. Finally, the raw sEMG signals were processed using the scientific Python development environment (SPYDER) (v.5.4.3, NumFOCUS, Austin, TX, USA) with a customized script that followed standard procedures [24]. A 4th order Butterworth filter was used, with a bandpass between 20 Hz and 500 Hz. The root mean square (RMS) was calculated for each muscle from a 5-s window in the middle of the 10-s recording for each CCFT level [22]. SEMG data were normalized and expressed as a percentage of the maximum RMS obtained during a 2-s MVIC for neck flexion and extension. The maximum RMS was determined using a 200 ms window (−100 ms and +100 ms) centered on each channel’s sEMG peak in the MVIC signal.

### 2.4. Statistical Analysis

We fitted a linear mixed model to the data using the glmer function from the lme4 package (v.1.1-35.3, R Core Team, Madison, WI, USA) in R (v.4.4.0, R Core Team, Vienna, Austria), specifying a gamma distribution with a log link function. The response variable was the normalized RMS of the sEMG. Fixed effects included group, moment, muscle, side, sex, and BMI. A random intercept was included for each subject to account for within-subject variability.

**Figure A1:**
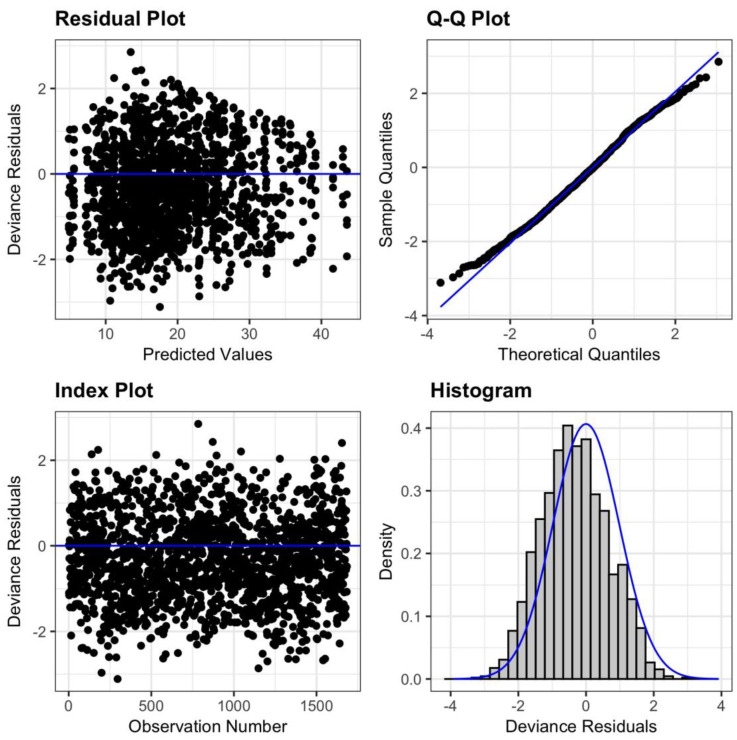
Linear mixed effects model diagnostics: Residual plot; Q−Q Plot; Index Plot; Histogram.

Model diagnostics (Figure A1) were performed using the resid_panel function from the ggResidpanel package (v.0.3.0, Iowa State University, IA, USA) to validate model assumptions and identify potential issues.

We calculated estimated marginal means for each combination of group, muscle, and moment using the emmeans function from the emmeans package (v.1.10.2, University of Iowa, IA, USA), applying asymptotic degrees of freedom. These results were back-transformed to the original scale by applying the inverse log transformation to the estimated marginal means and contrasts.

Pairwise contrasts were computed to evaluate the slope difference between endpoint and baseline moments for each group and muscle. Additional contrasts were calculated to compare slope differences between each muscle for control and neck pain groups.

## 3. Results

Sixty students were invited to participate in our study. Of these, three declined to participate. The remaining fifty-seven students agreed to participate and proceeded to data collection. During the study, thirteen (22.8%) participants dropped out after failing the second assessment, culminating in a final sample of forty-four participants. The participants were 68.2% women (*n* = 30) and 31.8% men (*n* = 14) with an average age of 22.34 ± 2.06 years and an average body mass index (BMI) of 21.76 ± 3.07 kg/m^2^. Participants were allocated into CG (*n* = 23) and NP groups (*n* = 21) based on the results of the NMQ. Detailed sociodemographic information on the participants is described in Table 1.

The developed linear mixed effects model was used to estimate the marginal means for the normalized RMS of the sEMG response, their standard errors, and 95% confidence intervals, as presented in Table 2. Model diagnostics are presented in Figure A1, located in Appendix A. The results are averaged over the levels of side and sex, and the values are back-transformed from the log scale into their original scale.

Pairwise contrasts were computed to compare the slope difference between the endpoint and baseline moments within each group and muscle. The results are presented in Table 3, with tests performed on the log scale. These results show that for the UT muscle, the sEMG response at the endpoint was significantly higher (1.64×) than at the baseline for the NP group (*p* < 0.001), but not for the CG (*p* = 0.980). In addition, results show that for the SCM muscle, the sEMG response at the endpoint was significantly lower than at the baseline for both the NP group (*p* = 0.038) (0.81×) and the CG (*p* < 0.001) (0.64×).

Additional contrasts were computed to compare each muscle’s slope differences between the CG and NP. These results are presented in Table 4, with tests performed on the log scale. These between-group contrasts indicated a significant difference in the slope changes between groups (1.65×) for the UT muscle (*p* < 0.001) but not for the SCM muscle (*p* = 0.097).

These findings suggest differential effects of the pain condition on sEMG responses depending on the muscle and the moment (endpoint vs. baseline).

## 4. Discussion

Cervical pain, despite its multifactorial nature [7,8], has been associated with changes in muscle strength [11,25,26,27] and motor unit activity [10,11,12,13,14,15], potentially contributing to the development of chronic musculoskeletal neck pain [12,16]. Increased activity of superficial flexors and extensors resulting from the functional adaptations to perform a low-load task [22,23] was previously observed in patients with neck pain. We hypothesized that a similar pattern could be found among dental students with neck pain. To improve our understanding of this issue, we investigated and compared the changes in cervical superficial muscle activation patterns between dental students with and without neck pain during their first semester of clinical training.

In our study, we observed a different dynamic within each group over the semester. Dental students with neck pain demonstrated increased UT activity during a low-load contraction task, specifically during the cranio-cervical flexion test. There was a 64% increase in UT activation in these students for the same task at the end of the first semester with clinical practice. This finding underscores the presence of altered neuromuscular control in dental students with neck pain, aligning with similar observations in other populations [9,10,11,12,13,14,15]. During low-load tasks like the CCFT, superficial flexor muscle activation is expected to be minimal because the deep neck flexors primarily perform the task and the superficial extensors have a subtle coactivation to stabilize the cervical spine [22,23]. Although group allocation divided students with neck pain from asymptomatic students, at the baseline, the groups had no differences regarding their cervical muscles’ activity pattern in flexors and extensors muscles. We expected that the ongoing exposure of these students to the physical demands of their clinical practice would manifest as increased coactivation of UT muscles in those with neck pain. Our findings confirmed the presence of this maladaptive change in the activation pattern of the neck extensors, aligning with previous reports in patients with neck pain [22,28]. Specifically, the activity of the UT in students with neck pain exceeded that of asymptomatic students, which remained similar to the baseline. Previous studies have shown that pain in the splenius capitis muscle results in reduced electromyographic activity during isometric cervical extension, coupled with increased activation of the trapezius muscle, which acts as a synergist for this movement [29], indicating that the compensatory strategies are dependent on the biomechanical constraints dictated by the task performed [6]. It suggests that, in response to a reflex inhibition of motor neurons innervating the painful muscle, the central nervous system appears to employ compensatory mechanisms that enable task execution, capitalizing on the system’s redundancy [6]. This could occur for the synergists to allow segment mobilization or for the antagonists to assist with segment stabilization [30]. In both cases, the increased activity of UT has been described as a common adaptation in individuals with neck pain. Therefore, the varying dynamics of the UT activation pattern observed between groups during the semester emphasize the complex interplay between pain, motor control, and compensatory strategies. Given that higher cervical loads require greater neuromuscular adaptations for task completion and enhanced cervical stability [30], the increased use of these muscles throughout the semester likely contributed to increased UT activation in students with neck pain. However, deficits in motor control of the spine can lead to inadequate control of joint movement, resulting in recurrent microtrauma and ultimately leading to pain [6]. Over time, increased activity in the UT, exacerbated by poor neck posture or awkward arm positions, may elevate compressive forces on the cervical segments, potentially leading to a painful neck condition [6]. Consequently, without appropriate intervention, this adaptation could be a provocative mechanism by altering load distributions and irritating sensitive structures, further exacerbating these adaptations and existing symptoms. This phenomenon has been observed in patients with chronic neck pain, where changes in sEMG activity are noted even during tasks that impose lower loads [22]. Ultimately, the increased coactivation of the neck extensors during the clinical practice tasks could result in a decreased capacity of strength production from neck flexors [22,31], creating an imbalance that could further exacerbate motor control deficits, neck symptoms, and dysfunction.

At the outset, we hypothesized that students with neck pain would exhibit heightened activity in superficial neck flexors, potentially as a compensatory mechanism for reduced activity in deep neck flexors, a pattern often seen in patients with neck pain [23]. However, contrary to our hypothesis, both groups had decreased SCM activation levels for the CCFT by the end of the semester. Given that the superficial flexor activity measured does not represent the actual agonist activity to perform the CCFT properly but rather compensatory activity during this low-load test [23], we can assume that the clinical practice of these students did not contribute to the development or enhance this compensatory pattern in low-load tasks. Instead, their clinical practice exposure had the opposite effect. The neuromuscular adaptation to clinical practice has maintained and reinforced the deep cervical flexors as the primary muscles for task execution, decreasing superficial neck flexor activity. Despite both groups having significant differences, the change in SCM activity showed a 36% decrease in the control group, almost doubling the 19% change observed in students with neck pain, compared to baseline levels, which could indicate that these groups might diverge in later stages of their clinical practice, replicating a previously observed pattern of increased SCM activity in students with pain [22,23], as we hypothesized. While the most plausible explanation for our findings is the neuromuscular adaptation to a new task, we cannot dismiss the possibility that this change may result from the learning effect of executing the CCFT during the second assessment.

In summary, the early stages of clinical practice influenced flexor function during the CCFT, as evidenced by a decrease in SCM activity in both groups, suggesting increased deep cervical flexor activity as expected for this task [22,23]. Despite this positive adaptation to clinical training, students with neck pain showed concomitant higher coactivation of the UT, presumably for enhanced cervical segment stability [30]. Contrary to our hypothesis of increased UT and SCM activity in students with neck pain following previous knowledge [9,10,11,12,13,14,15], our findings indicate that the neuromuscular adaptations from clinical training in cervical deep flexors, inferred from decreased SCM activity [22,23], overcame the expected superficial compensatory activation resulting in a decreased SCM activity in both groups. This adaptation was seen in both groups, reinforcing its association with clinical training. However, students with neck pain exhibited a smaller decrease in SCM activity than asymptomatic students, possibly due to UT coactivation. Still, it could also be an early indication of a possible divergent path in the later stages of clinical training following the changes observed in other populations [9,10,11,12,13,14,15].

The reported findings underscore the potential for targeted intervention development. Prior research has established that neuromuscular training involving low-load tasks and progressive load exposure without pain effectively diminishes neck pain and associated disability [32]. It also aids in modifying activation patterns to suit tasks [33,34], enhancing the activity of deep flexors while reducing the activity of superficial flexors and extensors coactivation. Nevertheless, future interventions or preventive measures should address the specific demand of dental clinical practice, which involves prolonged and sustained postures during patient care, since exercise programs are task-specific, and low-load training, such as using the CCFT modulates low-load performance [34], but not neck flexor strength and endurance performance at more intense contractions [35]. Intervention and prevention strategies derived from our findings should primarily focus on mitigating the increased activity of neck extensors, as this was identified as an early neuromuscular adaptation in these students.

While our study was designed to overcome the weaknesses of previous research, it still faced some limitations. Our methods, while based on prior studies on this topic, used an MVIC for normalization, which, in the presence of pain, could influence the participant’s capacity to fully activate their muscles while performing it because of the presence of confounding factors, such as kinesiophobia, which were not addressed and could affect the results. Future studies should consider using submaximal contractions for normalization to circumvent this issue. Additionally, several factors could influence the recorded sEMG activity, including task performance at two distinct times, background EMG activity, and the presence of artifacts, such as crosstalk [36]. Although the study’s methodology aimed to minimize these potential sources of interference, their complete exclusion from impacting the results cannot be guaranteed. Another limitation of our study is the unbalanced sex distribution in the neck pain group, which, despite reflecting the lower percentage of male students in the studied population and being considered in our linear mixed model for analysis, might have affected the outcomes. Future studies should explore the impact of sexual dimorphism on this matter and aim for larger sample sizes to enable sex-specific analyses. Finally, using a low-load task as the CCFT, with an assessment procedure differing from the working position, may not represent the regular demands during dental students’ clinical practice, but was selected due to its well-documented validity and reliability [37] and its successful use in previous research on neck flexors and extensors sEMG activity and their relation to pain [13,22,23].

Regarding the knowledge development on this issue, subsequent research efforts would benefit from introducing a control group not exposed to dental clinical practice to assess neuromuscular adaptations in neck pain and asymptomatic students exposed to this clinical setting. Another suggestion for future research could be to start the study by excluding students with existing neck pain and observe the students who develop it while enrolled in clinical practice, considering other associated variables. Future assessments of muscle activation patterns in specific clinical practice tasks and collecting EMG activity of deep flexor muscles could enhance our knowledge.

## 5. Conclusions

The present study confirmed that dental students exhibited decreased superficial flexor activity during the first semester of clinical training, transitioning to a more effective pattern that relies on deep flexors for a low-load task, such as the CCFT, as expected. However, students with neck pain exhibited a concomitant increased coactivation of the upper trapezius, presumably to enhance the stabilization of the painful cervical segment. This altered activation pattern emerged in symptomatic students when exposed to clinical practice and could be seen as a possible cause for further dysfunction and symptoms, potentially leading to chronic neck pain. These results should be considered to develop preventive and intervention exercise programs for dental students.

## Figures and Tables

**Table 1:** Sociodemographic characteristics of the participants.

	Control Group (*n* = 23)		Neck Pain Group (*n* = 21)		
	Mean ± SD	(Range)	Mean ± SD	(Range)	*p*
Age (years)	21.8 ± 1.92	(20–29)	22.9 ± 2.10	(21–30)	0.082 ^a^
Height (m)	1.72 ± 0.08	(1.58–1.88)	1.68 ± 0.10	(1.54–1.92)	0.180 ^a^
Body Mass (kg)	63.3 ± 10.65	(46–86)	63.5 ± 11.81	(47–88)	0.950 ^a^
BMI (kg/m^2^)	21.2 ± 2.11	(17.6–24.6)	22.4 ± 3.82	(16.9–35.3)	0.212 ^a^
Sex % (n)	Male 43.5% (10) Female 56.5% (13)		Male 19.1% (4) Female 80.9% (17)		0.082 ^b^

Note. BMI = Body Mass Index; SD = Standard Deviation; ^a^ Student’s *t*-test; ^b^ Chi-square test.

**Table 2:** Normalized RMS of the sEMG response by group, muscle, and moment.

Group	Moment	Muscle	% RMS	SE	95% Confidence Interval	
					Lower	Upper
Control	Baseline	UT	16.2	1.62	13.3	19.7
		SCM	24.4	2.52	19.9	29.9
	Endpoint	UT	16.2	1.62	13.3	19.7
		SCM	15.7	1.59	12.9	19.2
Neck Pain	Baseline	UT	13.3	1.51	10.7	16.6
		SCM	18.8	2.15	15.0	23.5
	Endpoint	UT	21.9	2.48	17.5	27.4
		SCM	15.3	1.74	12.2	19.1

Note. RMS = Root Mean Square; SCM = Sternocleidomastoid; SE = Standard Error; UT = Upper Trapezius.

**Table 3:** Pairwise contrasts for endpoint versus baseline moments.

Muscle	Group	Contrast	Ratio	SE	z.ratio	*p*
UT	Neck Pain	E/B	1.644	0.159	5.131	<0.001 *
	Control	E/B	0.998	0.092	−0.025	0.980
SCM	Neck Pain	E/B	0.812	0.081	−2.080	0.038 *
	Control	E/B	0.644	0.062	−4.537	<0.001 *

Note. B = Baseline; E = Endpoint; *p* = *p*-value; SCM = Sternocleidomastoid; SE = Standard Error; UT = Upper Trapezius; * = *p*-value < 0.05.

**Table 4:** Between-group contrasts for slope differences.

Muscle	Contrast	Ratio	SE	z.ratio	*p*
UT	(E/B) NP/(E/B) C	1.65	0.220	3.738	<0.001 *
SCM	(E/B) NP/(E/B) C	1.26	0.176	1.661	0.097

Note. B = Baseline; E = Endpoint; *p* = *p*-value; SCM = Sternocleidomastoid; SE = Standard Error; UT = Upper Trapezius; * = *p*-value < 0.05.

## Data Availability

The original data presented in the study are openly available in Mendeley Data at https://data.mendeley.com/datasets/g54r5t4gsp/1 (accessed on 31 May 2024).
